# Foramen magnum stenosis and midface hypoplasia in C-type natriuretic peptide-deficient rats and restoration by the administration of human C-type natriuretic peptide with 53 amino acids

**DOI:** 10.1371/journal.pone.0216340

**Published:** 2019-05-23

**Authors:** Takafumi Yotsumoto, Naomi Morozumi, Mayumi Furuya, Toshihito Fujii, Keisho Hirota, Yohei Ueda, Kazumasa Nakao, Shigeki Yamanaka, Kazunori Yoshikiyo, Sayaka Yoshida, Tomonari Nishimura, Yasuyuki Abe, Toshimasa Jindo, Hiroyuki Ogasawara, Akihiro Yasoda

**Affiliations:** 1 Asubio Pharma Co., Ltd. Kobe, Japan; 2 Daiichi Sankyo Co., Ltd. Tokyo, Japan; 3 Department of Diabetes, Endocrinology and Nutrition, Kyoto University Graduate School of Medicine, Kyoto, Japan; 4 Department of Oral and Maxillofacial Surgery, Kyoto University Graduate School of Medicine, Kyoto, Japan; 5 Clinical Research Center, National Hospital Organization Kyoto Medical Center, Kyoto, Japan; University of Cincinnati, UNITED STATES

## Abstract

C-type natriuretic peptide (CNP)-knockout (KO) rats exhibit impaired skeletal growth, with long bones shorter than those in wild-type (WT) rats. This study compared craniofacial morphology in the CNP-KO rat with that in the Spontaneous Dwarf Rat (SDR), a growth hormone (GH)-deficient model. The effects of subcutaneous administration of human CNP with 53 amino acids (CNP-53) from 5 weeks of age for 4 weeks on craniofacial morphology in CNP-KO rats were also investigated. Skulls of CNP-KO rats at 9 weeks of age were longitudinally shorter and the foramen magnum was smaller than WT rats. There were no differences in foramen magnum stenosis and midface hypoplasia between CNP-KO rats at 9 and 33 weeks of age. These morphological features were the same as those observed in CNP-KO mice and activated fibroblast growth factor receptor 3 achondroplasia-phenotype mice. In contrast, SDR did not exhibit foramen magnum stenosis and midface hypoplasia, despite shorter stature than in control rats. After administration of exogenous CNP-53, the longitudinal skull length and foramen magnum size in CNP-KO rats were significantly greater, and full or partial rescue was confirmed. The synchondrosis at the cranial base in CNP-KO rats is closed at 9 weeks, but not at 4 weeks of age. In contrast, synchondrosis closure in CNP-KO rats treated with CNP-53 was incomplete at 9 weeks of age. Administration of exogenous CNP-53 accelerated craniofacial skeletogenesis, leading to improvement in craniofacial morphology. As these findings in CNP-KO rats are similar to those in patients with achondroplasia, treatment with CNP-53 or a CNP analog may be able to restore craniofacial morphology and foramen magnum size as well as short stature.

## Introduction

C-Type natriuretic peptide (CNP), atrial natriuretic peptide (ANP), and brain natriuretic peptide (BNP) are structurally similar [1]. By using systemic or cartilage-specific CNP or natriuretic peptide receptor (NPR)-B knockout (KO) mice [2–6] and transgenic mice [7–10], we found that signaling by CNP and its receptor, NPR-B, is essential for endochondral bone growth.

We recently reported that CNP-KO rats also exhibited impaired skeletal growth, with long bones shorter than those in wild-type (WT) rats [11]. These rats lived over one year, and exhibited postnatal short stature and symmetric shortening of long bones, similar to CNP-KO mice [2]. In addition, early growth plate closure caused poor long bone growth, with final body length significantly less than that in WT rats. The phenotypes of CNP-KO [12] and activated fibroblast growth factor receptor 3 achondroplasia-phenotype (*Fgfr3*^*ach*^) mice [13] were similar to those in patients with achondroplasia, and the skull morphologies and narrow foramen magnum also resemble those in patients with achondroplasia [14].

This study compared craniofacial morphology in the CNP-KO rat with that in the Spontaneous Dwarf Rat (SDR), a growth hormone (GH)-deficient model [15]. We also administered human CNP with 53 amino acids (CNP-53) subcutaneously to CNP-KO rats from 5 weeks of age for 4 weeks, and investigated the restorative effect on craniofacial morphology. Furthermore, we conducted stereomicroscopic examination of the synchondrosis at the cranial base to determine the association with the restorative effect of CNP.

## Materials and methods

### Peptide

Human CNP-53 (GLSKGCFGLKLDRIGSMSGLGCKKNAGKYRANPHEQLLRA WAARSKTDVRLD) was produced by Asubio Pharma Co. Ltd., using a recombinant DNA method in *Escherichia coli*, purified with high-performance liquid chromatography, and verified by amino acid composition analysis and electrospray ionization mass spectrometry. The purity of CNP-53 was over 95%.

### Animals

CNP-KO rats were generated as previously described [11]. This study used 4-5-week-old, female, CNP-KO, homozygous Δ11 rats (at nucleotides 192–202, NM_053750.1) and littermate WT rats derived from F344/Stm rats deposited with the National Bio Resource Project for Rats in Japan. Seven-week-old female Sprague Dawley (SD) rats and SDRs, a dwarf strain derived from the SD rat, were purchased from Japan SLC, Inc. (Hamamatsu, Japan). The animals were housed in rooms with controlled humidity and temperature and 12-h lighting in the Kobe BM Laboratories of Oriental Bio-Service, Inc. They were fed a standard, pelleted lab chow diet (CRF-1, Oriental Yeast Co., Ltd., Japan) and tap water *ad libitum*. The rats were genotyped by quantifying the *Nppc* transgene using multicapillary electrophoresis (QIAxcel, QIAGEN). Animal care and experiments were conducted under the Guidelines for Animal Experiments of Oriental Bio-Service, Inc. and Asubio Pharma Co., Ltd., and all animal experiments were approved by the Animal Research Committee of Kyoto University, Institutional Animal Care and Use Committee of Oriental Bio-Service, Inc., and the Committees for Ethics in Animal Experiments of Asubio Pharma Co., Ltd.

### Experimental protocol

#### 1. Comparison of craniofacial morphology between CNP-KO rats and SDRs

Skulls from 9-week-old female CNP-KO rats (N = 4) and WT rats (N = 5) in the present experiment and from 33-week-old female CNP-KO rats (N = 3) and WT rats (N = 3) in a previous study [11] underwent imaging for craniofacial morphological analysis. Data of 9-week-old female SD rats and SDRs (each N = 5) were compared with data of CNP-KO rats; body weight and length were measured, and craniofacial morphology was analyzed with imaging as shown below.

#### 2. Craniofacial morphology in CNP-KO rats treated with CNP-53

Five-week-old female CNP-KO rats and WT rats received a continuous subcutaneous infusion of CNP-53 at an approximate dose of 0.5 mg/kg/day (CNP-KO rats, N = 5) or a vehicle (CNP-KO rats, N = 4; WT rats, N = 5) for 4 weeks, using an osmotic mini-pump (ALZET osmotic pump 2004, Durect Corporation, USA). CNP-53 was dissolved in 0.03 M acetate buffer (pH 4.0), 1% benzyl alcohol, and 10% purified sucrose to prepare the dosing solutions. We recently reported that continuous subcutaneous administration of CNP-53 significantly stimulated skeletal growth in CNP-KO rats at 0.5 mg/kg/day for 4 weeks [16]. The concentrations of the dosing solutions in this study were calculated based on an average body weight of CNP-KO rats and WT rats at 5 to 7 weeks of age in the present study and at 7 to 9 weeks of age in a previous study [16]. The initial dosing solutions were prepared for rats 5 to 7 weeks of age and the osmotic pumps were implanted for subcutaneous infusion. The pumps were replaced after 2 weeks with infusions of dosing solutions prepared for rats 7 to 9 weeks of age.

After a 4-week infusion, body weight and length were measured and craniofacial morphological analysis was performed with imaging and stereomicroscopic examination of the synchondrosis at the cranial base. Stereomicroscopic examination of the synchondrosis was also performed at 4 weeks of age in female CNP-KO and WT control rats (each N = 2), before the start of CNP-53 administration until 9 weeks of age in CNP-KO rats.

### Body weight and body length

Body weights were measured at 9 weeks of age in CNP-KO, WT, SDR, and SD rats. Body length was defined as the naso-anal length measured with a ruler under isoflurane inhalation anesthesia.

### Skull imaging

Isolated rat skulls were examined using computed tomography (CT) (Latheta LCT-200; Hitachi Aloka Medical, Japan). Parameters for CT were as follows: tube voltage, 50 kVp; tube current, 0.5 mA; integration time, 3.6 ms; axial field of view, 96 mm, with an isotropic voxel size of 96 μm. Reconstruction of 3-dimensional (3D) CT images and measurement of the length and area of the skull were performed using an image processing program (ImageJ, http://rsbweb.nih.gov/ij/download.html). The skull morphology (skull length, skull width, nose length, nasal bone length, inter-orbital distance, upper jaw length, and lower jaw length) was examined using linear measurements according to previously described methods [12, 17].

### Quantitative RT-PCR

Gene expression of matrix Gla protein (*Mgp*), progressive ankyloses protein (*Ank*) and nucleotide pyrophosphatase/phosphodiesterase 1 (*Npp1*) was evaluated in lumbar vertebra with the housekeeping gene hyproxanthine phosphoribosyltransferase 1 (*Hprt1*) by means of relative quantification by RT-PCR. Total RNA was extracted from the lumbar vertebra of 4 weeks of age in rats using RNeasy Lipid Tissue Mini Kit (Qiagen, Germany). Complimentary DNA was synthesized by reverse transcription reaction using ReverTra Ace (TOYOBO Co., Ltd., Japan). RT-PCR was performed with a StepOnePlus Real-time PCR System (Thermo Fisher Scientific, USA), using THUNDERBIRD SYBR qPCR MIX (TOYOBO Co., Ltd., Japan) and primers specific for *Mgp*, *Ank*, *Npp1* and *Hprt1* obtained from the manufacturer (Thermo Fisher Scientific, USA). Sequences of the PCR primer were shown in Table 1.

**Table 1:** Sequences of the PCR primer.

Gene	Forward primer sequence	Reverse primer sequence	Amplicon
*Mgp*	5'-CTTCACCACCCGGAGAAT-3'	5'-CTGCCTGAAGTAGCGGTTGT-3'	196-bp
*Ank*	5'- CATCACCAACATAGCCATCG -3'	5'- AAGGCAGCGAGATACAGGAA -3'	351-bp
*Npp1*	5'- GTCAGTATGCGTGCTAAC -3'	5'- TGGCACACTGAACTGTAG -3'	309-bp
*Hprt1*	5'- CTCATGGACTGATTATGGACAGGAC -3'	5'- GCAGGTCAGCAAAGAACTTATAGCC -3'	123-bp

### Stereomicroscopic examination of the synchondrosis at the cranial base

CNP-KO and WT rats at 4 weeks of age and CNP-KO and WT rats at 9 weeks of age after a 4-week infusion were sacrificed and decapitated. After removal of the head skin, isolated skull bones were fixed in 10% neutral buffered formalin solution and post-fixed in 95% ethanol solution. The skull bone was stained with alcian blue and alizarin red, and soft tissue was dissolved using 2% KOH solution. The spheno-occipital synchondrosis (SOS) at the cranial base was examined with stereomicroscopy.

### Statistical analysis

All data were expressed as means ± standard deviation (SD). Statistical analysis was performed using Student’s *t*-test by the statistical analysis system. *P* values less than 0.05 were considered statistically significant.

## Results

### Growth and development in CNP-KO rats and SDRs

**Fig 1:**
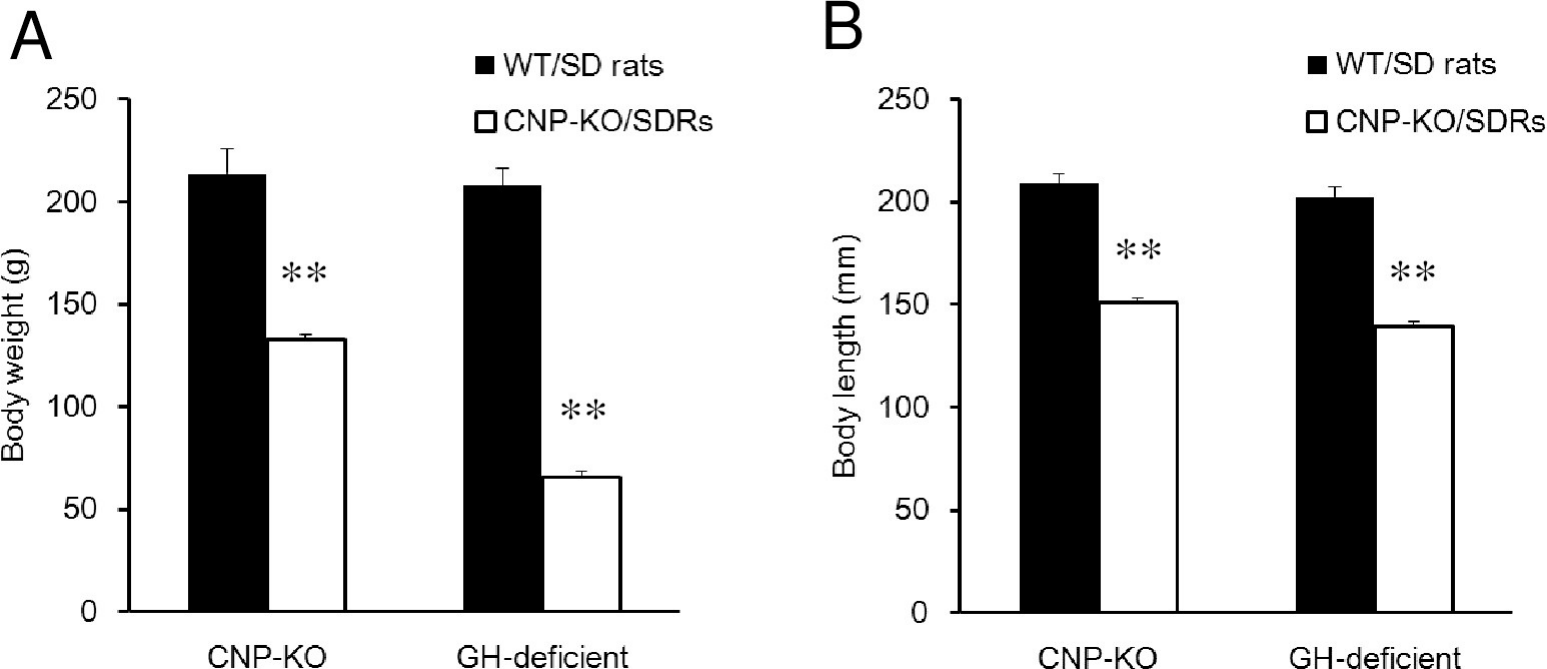
Weights and lengths of WT, CNP-KO, SD rats and SDRs. (A) Body weights and (B) lengths of female WT, CNP-KO, SD rats, and SDRs at 9 weeks of age. Body weights and lengths of WT and CNP-KO rats at 9 weeks of age (N = 3 each), and of SD rats and SDRs at 9 weeks of age (N = 5 each). Data are expressed as the means ± SD. **, *P* < 0.01 vs. WT or SD rats using Student’s *t*-test.

CNP-KO rats and SDRs had lower weights and shorter lengths than WT rats and normal SD rats at 9 weeks of age (Fig 1). The body weights and lengths of WT rats and CNP KO rats in this study showed the similar results with previous study [16]. SDRs weighed 31.6% less and were 68.9% shorter than control SD rats, and CNP-KO rats weighed 77.3% less and were 68.6% shorter than WT rats at 9 weeks of age.

### Morphological analyses of CNP-KO and SDR skulls

**Fig 2:**
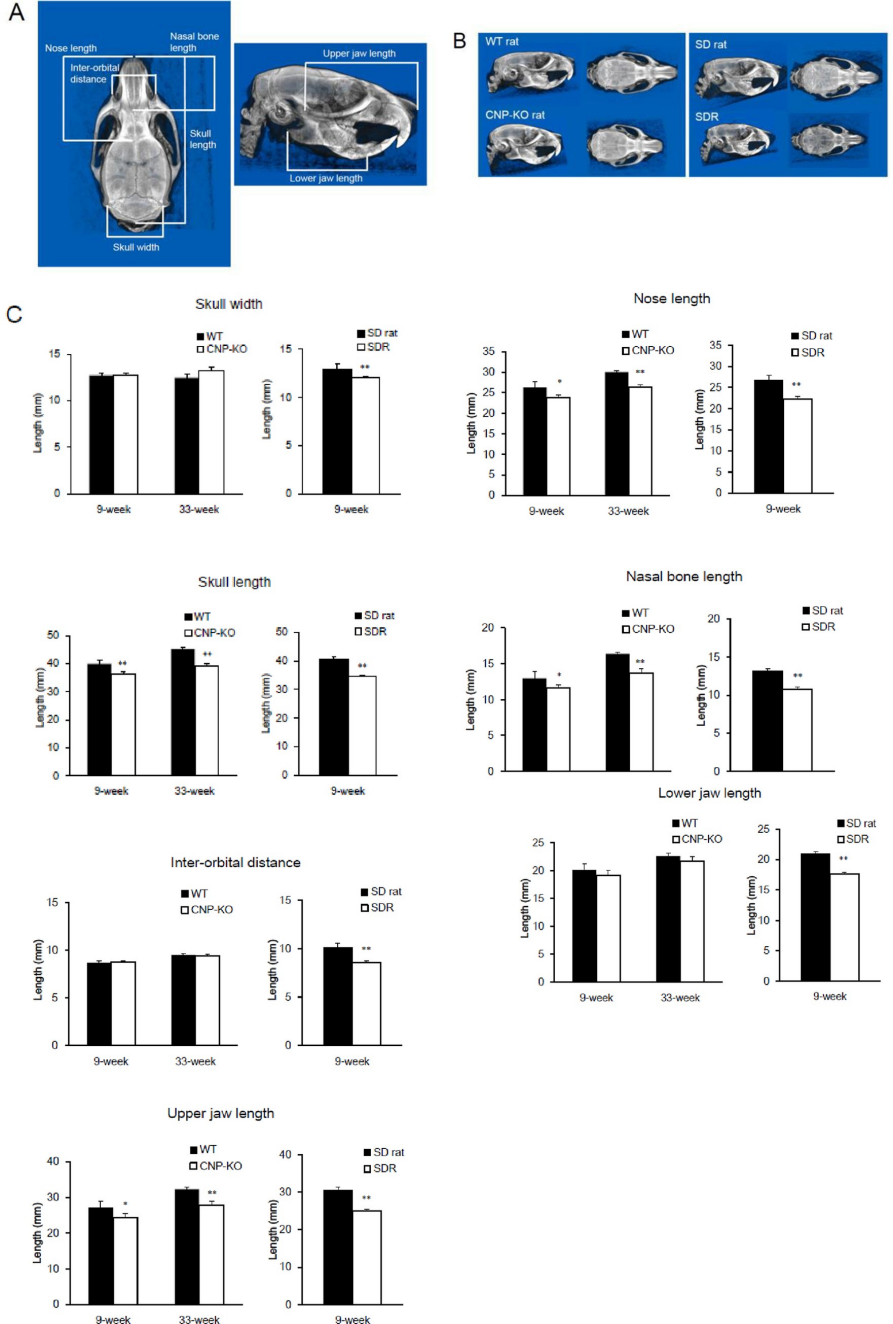
Analyses of craniofacial morphology in WT, CNP-KO, SD rats, and SDRs. (A) Linear measurements for analysis of skull morphology. (B) 3D-reconstructed images of WT, CNP-KO, SD rats, and SDRs at 9 weeks of age. (C) Linear measurements of craniofacial morphology of WT and CNP-KO rats at 9 and 33 weeks of age (N = 3 each), and of SD rats and SDRs at 9 weeks of age (N = 5 each). Data are expressed as the means ± SD. *, *P* < 0.05, **, *P* < 0.01 vs. WT or SD rats using Student’s *t*-test.

Skull length, skull width, nose length, nasal bone length, inter-orbital distance, upper jaw length, and lower jaw length were determined using linear measurements in CNP-KO and SDRs (Fig 2A). Reconstructed 3D CT images were used for morphological analyses (Fig 2B). The skull length, nose length, nasal bone length, and upper jaw length in CNP-KO rats were significantly shorter than those in WT rats, while the skull width and inter-orbital distance in CNP-KO rats were almost the same as those in WT rats (Fig 2C). These results showed that the skulls of CNP-KO rats were longitudinally shorter than those of WT rats. A longitudinally short skull was also observed at 33 weeks of age in CNP-KO rats. In contrast, all measurements for morphological analysis were significantly shorter in SDRs than in SD rats, and SDR did not exhibit a longitudinally shorter phenotype.

### Foramen magnum size in CNP-KO rats and SDRs

**Fig 3:**
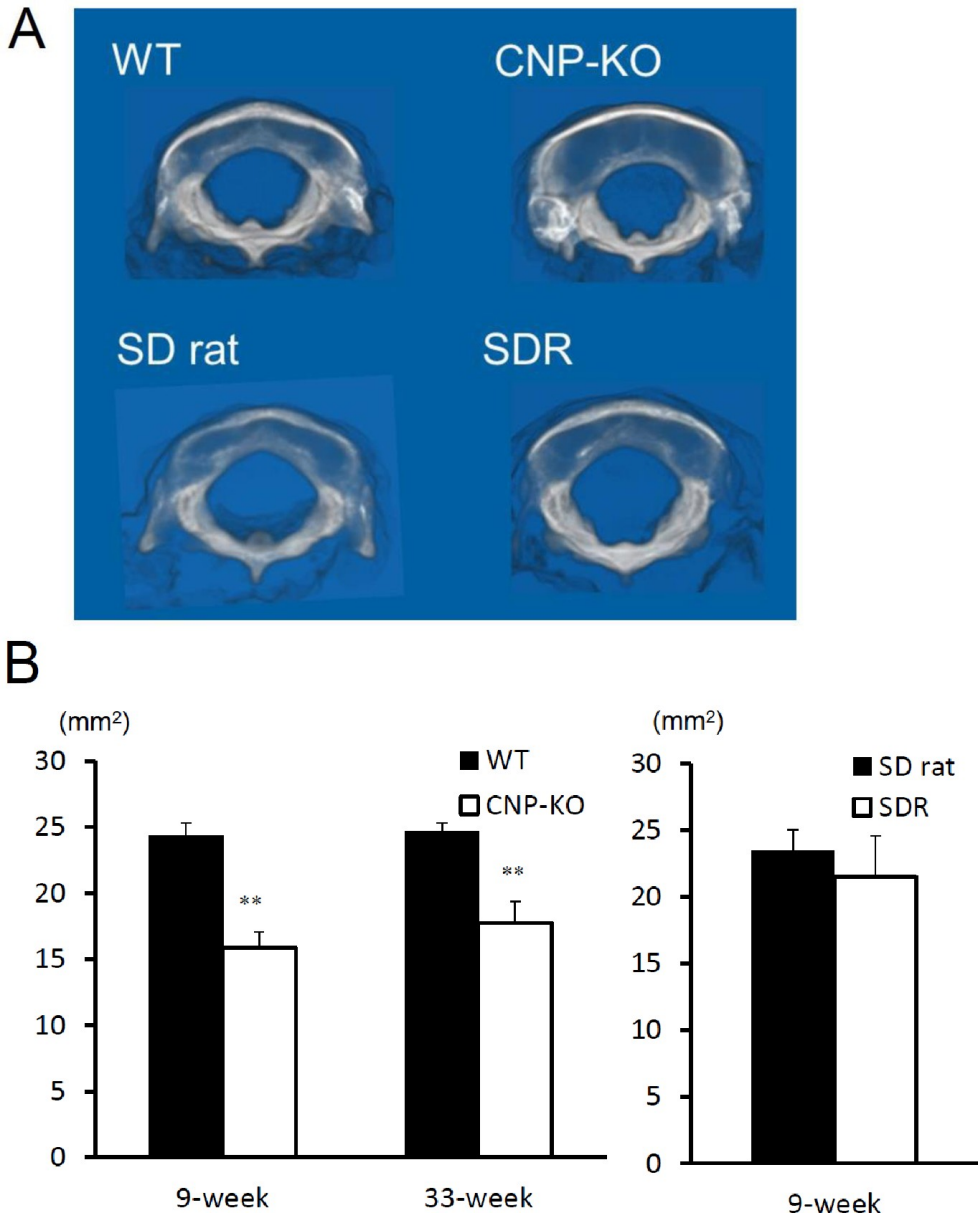
Foramen magnum size in WT, CNP-KO, SD, and SDR rats. (A) 3D-reconstructed images of the foramen magnum in WT and CNP-KO rats at 9 weeks of age, and in SD rats and SDRs at 9 weeks of age. (B) Foramen magnum size in WT and CNP-KO rats at 9 and 33 weeks of age (N = 3 each), and in SD rats and SDRs at 9 weeks of age (N = 5 each). Data are expressed as the means ± SD. *, *P* < 0.05, **, *P* < 0.01 vs. WT or SD rats using Student’s *t*-test.

The foramen magnum was measured using reconstructed 3D CT images (Fig 3A). The foramen magnum size was significantly smaller in CNP-KO rats compared with WT rats at both 9 and 33 weeks of age. Between 9 and 33 weeks of age, the sizes of foramen magnum were similar in both CNP-KO and WT rats. In contrast, little difference was observed in foramen magnum size between SD rats and SDRs despite a large difference in the body weight and length.

### Effects of CNP-53 on skull morphology and development of the foramen magnum

**Fig 4:**
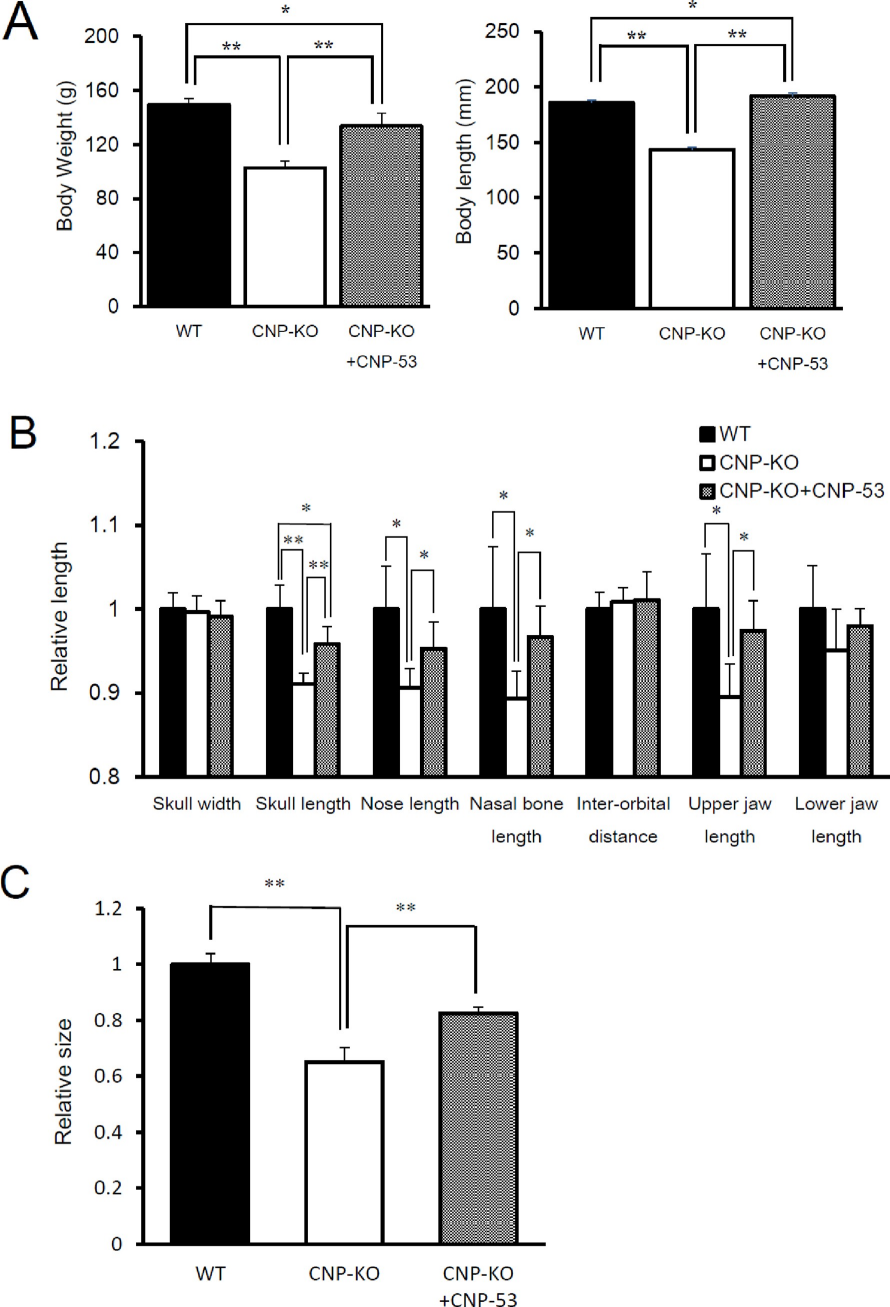
Effects of CNP-53 on body weight, length, craniofacial morphology, and foramen magnum size in CNP-KO rats. (A) Body weight and length, (B) relative lengths of craniofacial morphology to WT rats, (C) relative size of foramen magnum to WT rats. WT, WT rats which received vehicle; CNP-KO, CNP-KO rats which received vehicle; CNP-KO + CNP, CNP-KO rats which received CNP-53 (ca. 0.5 mg/kg/day). Data are expressed as the means ± SD of 4–5 rats. *, *P* < 0.05, **, *P* < 0.01 vs. WT or CNP-KO rats using Student’s *t*-test.

Female CNP-KO and WT rats received CNP-53 at an approximate dose of 0.5 mg/kg/day for 4 weeks under the same conditions with our previous study [16]. After 4 weeks of administration, body weights and lengths in CNP-KO rats were significantly greater than those in control CNP-KO rats (Fig 4A). Linear measurements of skull morphology and foramen magnum size were concurrently performed using the reconstructed 3D CT images. Significantly greater values were observed in skull length, nose length, nasal-orbital length, and upper jaw length following CNP-53 administration. These measurements increased to approximately the same values as those in WT rats (Fig 4B). Although the foramen magnum enlarged significantly with CNP-53 administration in CNP-KO rats, the size did not reach that of WT rats (Fig 4C).

### Stereomicroscopic examination of the synchondrosis at the cranial base

**Fig 5:**
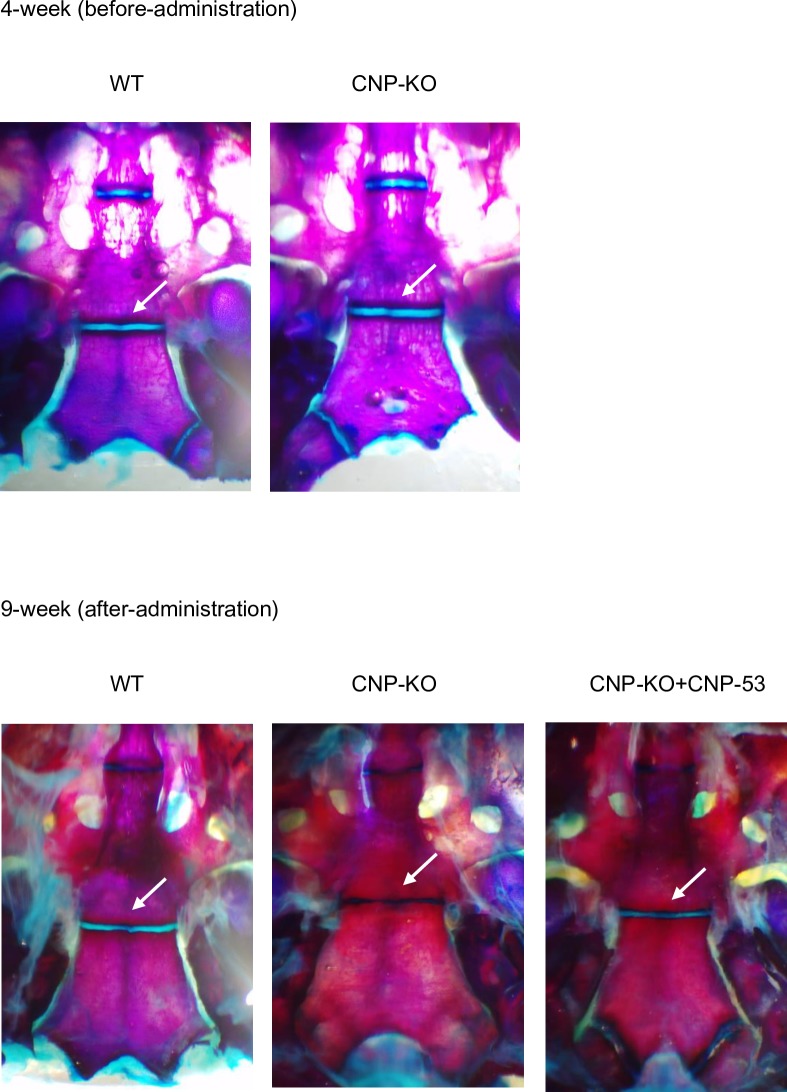
Appearance of the synchondrosis at the cranial base after staining with alcian blue and alizarin red. Arrow indicates the SOS.

The above results indicated the existence of midfacial hypoplasia in CNP-KO rats, and we further examined the SOS of CNP-KO rats. The effect of treatment with CNP-53 on synchondrosis at the cranial base was also evaluated. The SOS was assessed in both WT and CNP-KO rats at 4 weeks of age. At 9 weeks of age after 4 weeks of administration, the SOS was also assessed in both WT and CNP-KO rats treated with CNP-53. The SOS was closed in CNP-KO rats at 9 weeks of age (Fig 5).

### Craniosynostosis and nasal septum calcification in CNP-KO rats

In order to further investigate the possible midfacial hypoplasia observed in CNP-KO rats, we checked the presence of craniosynostosis in rats: using 3D CT images of whole-mount skull, lateral cephalic and magnification of lambdoidal and coronal were evaluated. We did not observe craniosynostosis in both WT and CNP-KO rats at 9 weeks of age (S1 Fig). Moreover, nasal septum calcification was not detected in CNP-KO rats as well as in WT rats at 9 weeks of age (S1 Fig).

### The expression of genes relevant to midfacial hypoplasia in the skeletal tissue of CNP-KO rats

Finally, we estimated mRNA levels of *Mgp*, *Ank* and *Npp1*, which were reported to be causative for midfacial hypoplasia [18], in lumber vertebrae of CNP-KO rats at 4 weeks of age. Alterations of mRNA level of *Mgp*, *Ank* and *Npp1* in lumbar vertebra of CNP-KO rats at 4 weeks of age were investigated. The mRNA levels of *Mgp* in CNP-KO rats were significantly lower than those of WT rats. *Ank* and *Npp1* levels tended to be lower, but not significant (S2 Fig).

## Discussion

The present study investigated the growth and development of skull morphology and foramen magnum size using specimens from CNP-KO rats at 9 and 33 weeks of age, and compared these parameters with those in SDRs, which are GH-deficient. The skull was longitudinally shorter and the foramen magnum was smaller in CNP-KO rats than in WT rats; these changes were not observed in SDRs.

We recently reported that CNP-KO rats exhibited impaired skeletal growth, with long bones shorter and growth plates narrower than those in WT rats [11], consistent with findings in CNP-KO mice and *Fgfr3*^*ach*^ mice [2, 19]. CNP-KO rats survived more than 1 year, while about 70% of CNP-KO mice died before 10 weeks of age [2, 11]. The craniofacial morphological features observed in CNP-KO rats were the same as those in CNP-KO mice and *Fgfr3*^*ach*^ mice [12, 17], and were similar to those in patients with achondroplasia [14]. In contrast, longitudinally short skulls and small foramen magnum size were not apparent in SDRs, despite shorter stature compared to control SD rats. Similarly, foramen magnum stenosis is not observed in GH-deficient patients [20], indicating that CNP/NPR-B signaling is involved in endochondral ossification at the skull base related to longitudinal growth and foramen magnum size. There were no age-related differences in longitudinal skull morphology and foramen magnum size between WT and CNP-KO rats, in which foramen magnum enlargement had almost ended by 9 weeks of age.

Midface hypoplasia and premature closure of the SOS were also reported in a mouse model lacking *Mgp* [18]. Considering the similar traits in the mutants, we investigated the alterations of mRNA level of *Mgp*, *Ank* and *Npp1* in lumbar vertebra and whether the CNP-KO rats show craniosynostosis and nasal septum calcification at 9 weeks of age. *Mgp*, *Ank* and *Npp1* are known as the mineralization inhibitor regulating genes, and craniosynostosis and nasal septum calcification are other features exhibiting in a mouse model lacking matrix Gla protein [18]. The mRNA levels of *Mgp* in CNP-KO rats were significantly lower than those of WT rats, and *Ank* and *Npp1* tended to be lower. Meanwhile, craniosynostosis and nasal septum calcification were not seen in CNP-KO rats. *Mgp* mRNA is expressing in resting, proliferative and late hypertrophic chondrocytes in growth plate cartilage [21]. The lower levels of *Mgp*, *Ank* and *Npp1* in CNP-KO rats might reflect the outcome of the decrease in endochondral ossification in these rats. Collectively, calcification of bones relevant to Mgp pathways in CNP-KO rats might be accelerated to some extent, which is in accordance with the result of our previous report showing an accelerated ossification in the growth plates in elder CNP-KO rats [16].

We investigated the effects of CNP-53 treatment from 5 weeks of age on skeletogenesis, skull morphology, and foramen magnum size. CNP-53 was administered at an approximate dose of 0.5 mg/kg/day for 4 weeks as a continuous subcutaneous infusion. As a result, the longitudinal skull measurement and foramen magnum size were significantly enlarged, and full or partial rescue was confirmed with CNP-53 treatment from 5 weeks of age. Yamanaka et al. previously reported the potential effect of circulating CNP on craniofacial hypoplasia using *Fgfr3*^ach^/SAP-*Nppc*-Tg mice, crossed *Fgfr3*^*ach*^ mice with transgenic mice in which CNP is expressed in the liver under the control of the human serum amyloid-P component promoter, resulting in elevated levels of circulatory CNP [17]; this is the first report that demonstrate the therapeutic potential of CNP for the treatment of foramen magnum stenosis and midfacial hypoplasia in achondroplasia. The developmental mechanisms of midfacial hypoplasia and foramen magnum stenosis in achondroplasia are related to FGFR3 and mitogen-activated protein kinase (MAPK) signaling in chondrocytes, which regulates synchondrosis closure, osteoblast differentiation, and bone formation [13]. FGFR3 is a key negative regulator of endochondral bone growth and signals mediate several intracellular pathways, including those of the signal transducer and activator of transcription (STAT) and MAPK [22]. The MAPK signaling pathway is also regulated by CNP [7], i.e., the binding of CNP to NPR-B inhibits FGFR3 downstream signaling at Raf-1 [23]. It was suggested that these results using CNP-KO rats would be replicated in *Fgfr3*^*ach*^ mice based on the results of *Fgfr3*^ach^/SAP-*Nppc*-Tg mice and mechanistic information.

Meanwhile, several other groups failed to restore foramen magnum size with administration of meclizine and BMN-111, an analogue of CNP, using *Fgfr3*^*ach*^ mice [24, 25]. The SOS in *Fgfr3*^*ach*^ mice was closed at 10 days of age [13], therefore the cause of their failure was thought to be premature synchondrosis closure in *Fgfr3*^*ach*^ mice. In contrast, the SOS of CNP-KO rats was observed at 4 weeks of age, one week before the start of CNP-53 administration. As a result, exogenous CNP-53 could restore foramen magnum size of CNP-KO rats in our study. The SOS in humans can be assessed using radiographic examination, with normal closure between 11 and 25 years of age [26–28]. These findings suggest that exogenous administration of CNP-53 may be able to restore craniofacial morphology and foramen magnum size in the patients whose SOS at the cranial base is still present as well as short stature in individuals with a growth disorder. At the end of CNP-53 administration at 9 weeks of age, the SOS was observed in both WT and CNP-KO rats treated with CNP-53, but was already closed in CNP-KO rats. Thus, CNP-KO rats exhibited early SOS closure, and exogenous CNP-53 prevented closure of the SOS as well as the growth plate in the femur and tibia [16].

In summary, CNP-KO rats had foramen magnum stenosis and midface hypoplasia, while these changes were not present in SDRs. The administration of CNP-53 improved foramen magnum stenosis and midface hypoplasia in CNP-KO rats. These features in CNP-KO rats were similar to those in patients with achondroplasia, and that CNP-53 or CNP analog therapy has the potential to restore craniofacial morphology and correct short stature.

## Supporting information

S1 Fig Craniosynostosis and nasal septum in WT and CNP-KO rats. (PPTX)

S2 Fig Comparison of gene expression of *Mgp*, *Ank* and *Npp1* between WT and CNP-KO rats. (PPTX)

S1 Table Individual value of all figures in this report. (XLSX)
