# Supplementary material for: Foramen magnum stenosis and midface hypoplasia in C-type natriuretic peptide-deficient rats and restoration by the administration of human C-type natriuretic peptide with 53 amino acids
Source: PLoS One. 2019 May 23;14(5):e0216340. doi: 10.1371/journal.pone.0216340 (PMC6532844; doi:10.1371/journal.pone.0216340)
Supplement: S1 Fig — (PPTX) [file pone.0216340.s001.pptx]

## Slide 1
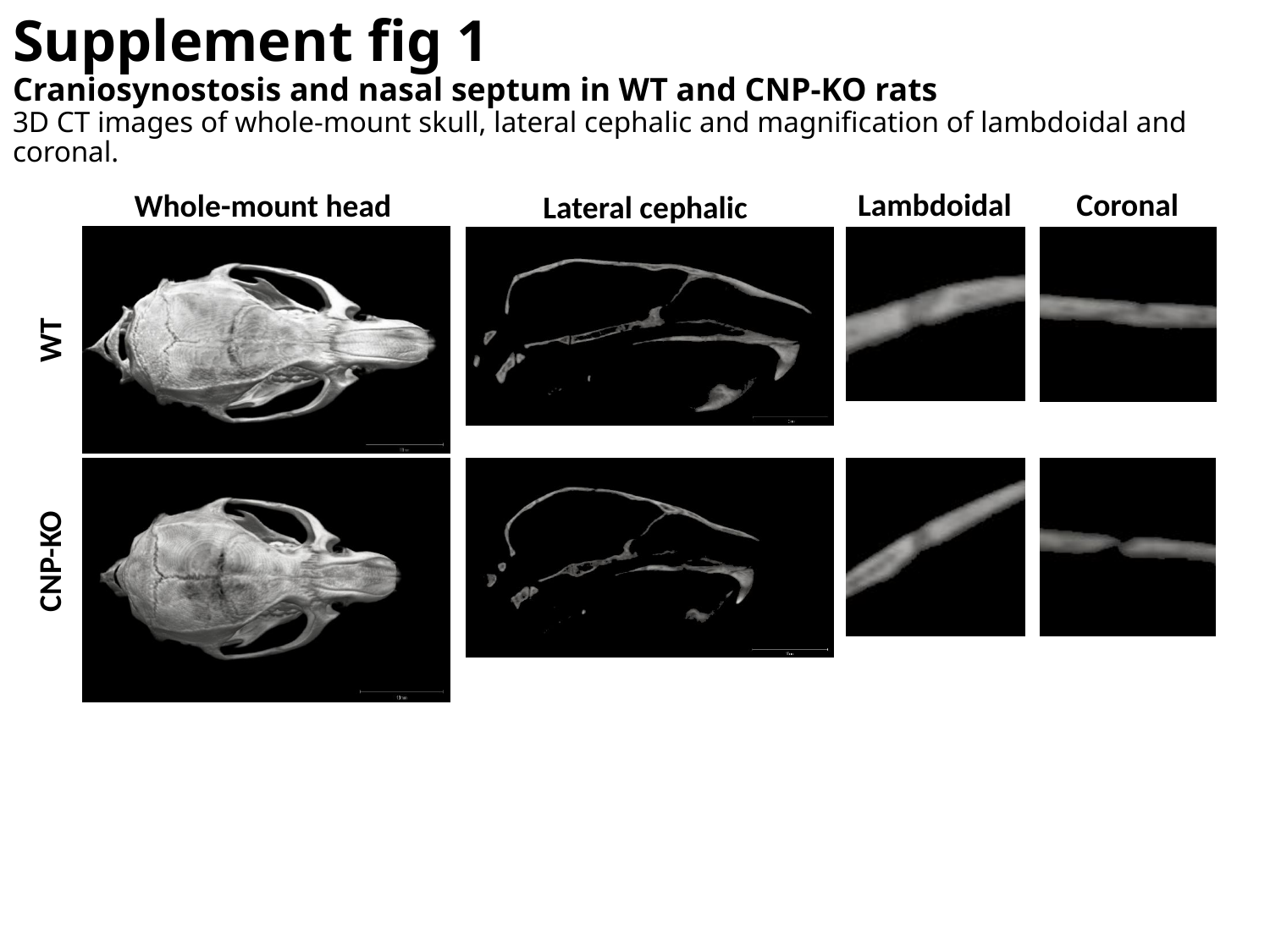

# Supplement fig 1 Craniosynostosis and nasal septum in WT and CNP-KO rats 3D CT images of whole-mount skull, lateral cephalic and magnification of lambdoidal and coronal.
Lambdoidal
Coronal
Whole-mount head
Lateral cephalic
WT
CNP-KO
