# Supplementary material for: Foramen magnum stenosis and midface hypoplasia in C-type natriuretic peptide-deficient rats and restoration by the administration of human C-type natriuretic peptide with 53 amino acids
Source: PLoS One. 2019 May 23;14(5):e0216340. doi: 10.1371/journal.pone.0216340 (PMC6532844; doi:10.1371/journal.pone.0216340)
Supplement: S2 Fig — (PPTX) [file pone.0216340.s002.pptx]

## Slide 1
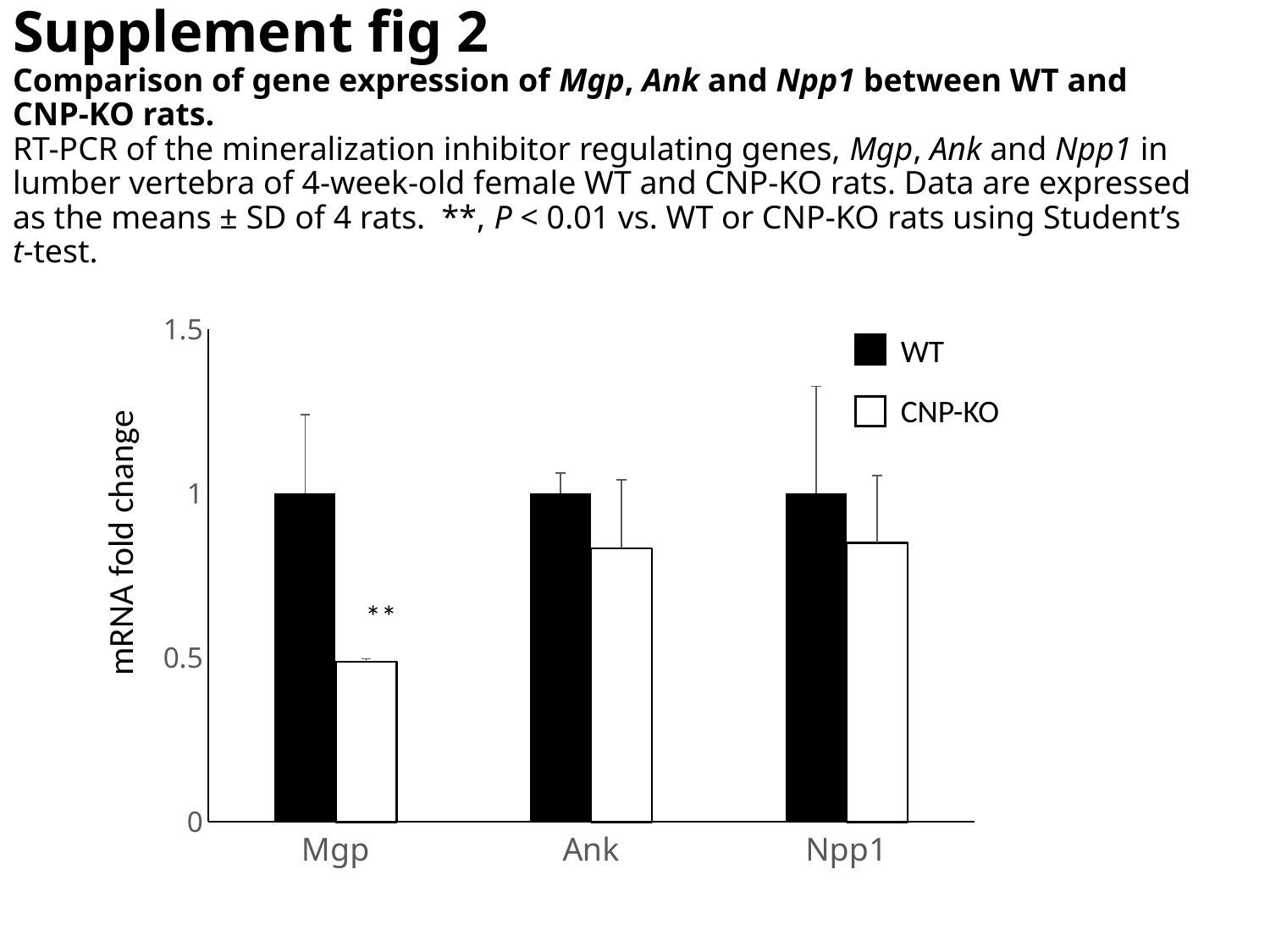

# Supplement fig 2 Comparison of gene expression of Mgp, Ank and Npp1 between WT and CNP-KO rats. RT-PCR of the mineralization inhibitor regulating genes, Mgp, Ank and Npp1 in lumber vertebra of 4-week-old female WT and CNP-KO rats. Data are expressed as the means ± SD of 4 rats. **, P < 0.01 vs. WT or CNP-KO rats using Student’s t-test.
### Chart
| Category | WT | KO |
|---|---|---|
| Mgp | 1.0 | 0.48773918878856504 |
| Ank | 1.0 | 0.8328786267145954 |
| Npp1 | 1.0 | 0.8497698336612742 |WT
CNP-KO
mRNA fold change
**
